# Effects of Decayed Teeth

**Published:** 1857-10

**Authors:** J. S. Dixon

**Affiliations:** Ashland, Tenn.


### ARTICLE III.

*Effects of Decayed Teeth.* By J. S. Dixon, Ashland, Tenn.

I may be thought egotistic in publishing my own case, but as a man can describe his own feelings better than another's, these are, I think, the cases that should be preferred for publication.

I am aged thirty-three. During the years 1855-'56, I was in very delicate health, and several of my teeth became badly affected, though they seldom ached. In 1855 I was frequently affected with a severe neuralgia, or severe pain over my left eye, and the left side of my nose became affected with a constant dripping, which was very unpleasant, and caused me to fear ozæna; this became much worse, and during the last winter I was obliged to remove large plugs of hardened pus and mucus every day with a pair of forceps; this pressed the septum narium greatly to the right side. During the latter part of the winter, my fauces became badly inflamed and ulcerated, and resisted every means of cure, including caustic gargles, tonics, &c. In April the neuralgia returned more severely than ever, and resisted every known means of treatment. I then had all my carious teeth extracted by Dr. J. T. Collier, of this county, when my throat immediately commenced improving, and the discharge from my nose began to subside, since which I have had but one slight attack of neuralgia ; my throat is well now; the discharge has nearly ceased, and my general health has greatly improved, and I have used no medicine since the teeth were extracted. My bowels are now regular, and my appetite good, which has not been the case for the last two years. The septum narium still remains so much to the right of the median line as almost to occlude the right passage. I intend to try to rectify this with ents.

